# Evaluation and Structure-Activity Relationship Analysis of a New Series of *Arylnaphthalene lignans* as Potential Anti-Tumor Agents

**DOI:** 10.1371/journal.pone.0093516

**Published:** 2014-03-27

**Authors:** Jiaoyang Luo, Yichen Hu, Weijun Kong, Meihua Yang

**Affiliations:** Institute of Medicinal Plant Development, Chinese Academy of Medical Sciences and Peking Union Medical College, Beijing, P.R. China; Indian Institute of Toxicology Research, India

## Abstract

Arylnaphthalene lignan lactones have attracted considerable interest because of their anti-tumor and anti-hyperlipidimic activities. However, to our knowledge, few studies have explored the effects of these compounds on human leukemia cell lines. In this study, five arylnaphthalene lignans including 6′-hydroxy justicidin A (HJA), 6′-hydroxy justicidin B (HJB), justicidin B (JB), chinensinaphthol methyl ether (CME) and Taiwanin E methyl ether (TEME) were isolated from *Justicia procumbens* and their effects on the proliferation and apoptosis of the human leukemia K562 cell line were investigated then used to assess structure-activity relationships. To achieve these aims, cytotoxicity was assayed using the MTT assay, while intracellular SOD activity was detected using the SOD Activity Assay kit. Apoptosis was measured by both the using a cycle TEST PLUS DNA reagent kit as well as the FITC Annexin V apoptosis detection kit in combination with flow cytometry. Activation of caspase-mediated apoptosis was evaluated using a FITC active Caspase-3 apoptosis kit and flow cytometry. The results indicated that HJB, HJA and JB significantly inhibited the growth of K562 cells by decreasing both proliferation and SOD activity and inducing apoptosis. The sequence of anti-proliferative activity induced by the five tested arylnaphthalenes by decreasing strength was HJB > HJA > JB > CME > TEME. HJB, HJA and JB also decreased SOD activity and induced apoptosis in a dose-dependent manner. Activation of caspase-3 further indicated that HJB, HJA and JB induced caspase-dependent intrinsic and/or extrinsic apoptosis pathways. Together, these assays suggest that arylnaphthalene lignans derived from *Justicia procumbens* induce apoptosis to varying degrees, through a caspase-dependent pathway in human leukemia K562 cells. Furthermore, analysis of structure-activity relationships suggest that hydroxyl substitution at C-1 and C-6′ significantly increased the antiproliferative activity of arylnaphthalene lignans while a methoxyl at C-1 significantly decreased the effect.

## Introduction

Lignans are a large group of dimeric phenylpropanoids that are widely distributed in higher plants. Like many other secondary metabolites, lignans represent a means of protection against herbivores for the plants that synthesize them. There is a growing interest in exploiting lignans, and their synthetic derivatives, as potential anti-cancer agents [1], [2]. Indeed, some cytotoxic lignan derivatives have reached phase I and II clinical trials as anti-tumor agents including GP-11 [3], NK-611 [4], [5], TOP-53 [6], NPF [7], GL-331 [8]–[12]. More recently, the lignan F11782 was shown to be a novel catalytic inhibitor of topoisomerases I and II, key promoters of DNA replication [13].

The majority of natural arylnaphthalene lignans are lactones [14]. These have attracted considerable interest because of their anti-tumor and anti-hyperlipidemic activities [15]. Cytotoxicity as well as anti-cancer activity has been reported for arylnapthalene lignan isolated from several genus including *Phyllantus*, *Cleistanthus*, and *Justicia* [1], [16], [17].

*Justicia procumbens* (*J. procumbens*) is an herb which can be used to prepare a traditional remedy for the treatment of fever, pain, and cancer [18]. The bioactive justicins isolated from *J. procumbens* include diphyllin, 6′-hydroxy justicidin A (HJA) and chinensinaphthol methyl ether (CME), which share a similar chemical structure with that of podophyllotoxin (POD). Previous reports have demonstrated that these extracts promote cytotoxicity [19]–[23], antimicrobial [24], antiviral [25], and anti-platelet [26] activities. Indeed, the cytotoxicity of these arylnaphthalene lignans has been demonstrated in liver cancer HepG2, breast cancer MCF-7, lymphocytic leukemia P338 tumor cell lines, as well as human bladder cancer EJ cells. [18]–[20], [27].

We have also previously shown that the extract of *J. procumbens* displays broad-spectrum anti-tumor activity, especially in the human leukemia K562 cell line. In addition, we have isolated five arylnaphthalene lignans from *J. procumbens* including HJA, 6′-hydroxy justicidin B (HJB), justicidin B (JB), CME and Taiwanin E methyl ether (TEME) [28], [29] and demonstrated that these are the main arylnaphthalene lignans in *J. procumbens* [30]. Notably, these compounds share the same parent nucleus but harbor different substituent groups at the 1, 3′, 4′ and 6′ positions. However, the anti-cancer activity of these *J. procumbens*-derived compounds and their underlying mechanisms of action, especially the analysis of structure-activity relationships, have not been fully elucidated.

Mitochondria play a central role in various pathophysiological processes of cancer cells, in particular apoptosis. Most anti-tumor drugs can induce apoptosis in different types of tumor cells. There are two major apoptotic pathways, the intrinsic pathway and the extrinsic pathway, which both result in activation of effector caspases. The intrinsic apoptotic pathway involves an increase in mitochondrial membrane permeability and the subsequent increased release of cytochrome c into the cytoplasm, which in turn activates caspase-9 and caspase-3. These in turn mediate apoptotic damage [31], [32]. The extrinsic pathway is initiated by the activation of death receptors involves the formation of a death-inducing signaling complex (DISC). DISC formation results in the activation of caspase-8, which activates caspase-3 [33]. In addition, reactive oxygen species (ROS), a by-product of mitochondrial oxidative metabolism, have been reported to exert a pathogenic role in different degenerative diseases as well as cancer [34], [35].

**Figure 1:**
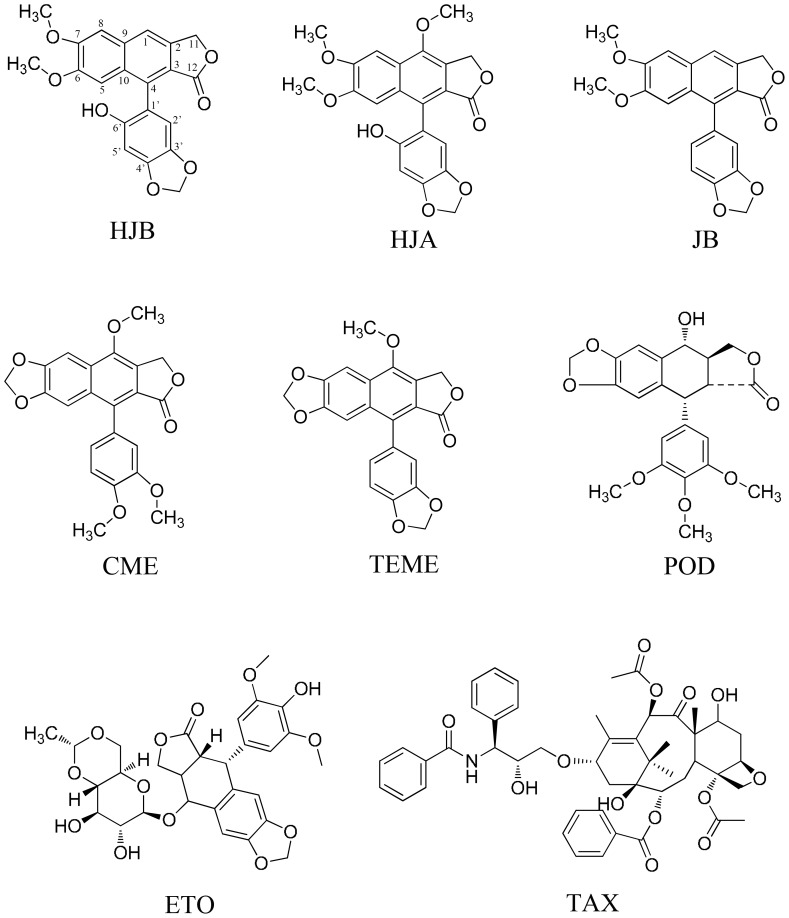
Chemical structures of 6′-hydroxy justicidin B (HJB), 6′-hydroxy justicidin A (HJA), justicidin B (JB), chinensinaphthol methyl ether (CME), Taiwanin E methyl ether (TEME), etoposide (ETO), paclitaxel (TAX) and podophyllotoxin (POD).

In the present study, we investigated whether, and to what degree, arylnaphthalene lignans affect the survival of the human leukemia K562 cell line by measuring viability and apoptosis after exposure to the five compounds (Figure 1). We then used these results to clarify the structure-activity relationship between arylnaphthalene lignans and their anti-cancer activity.

## Results

### Effect of arylnaphthalene lignans on leukemia K562 cell survival

**Figure 2:**
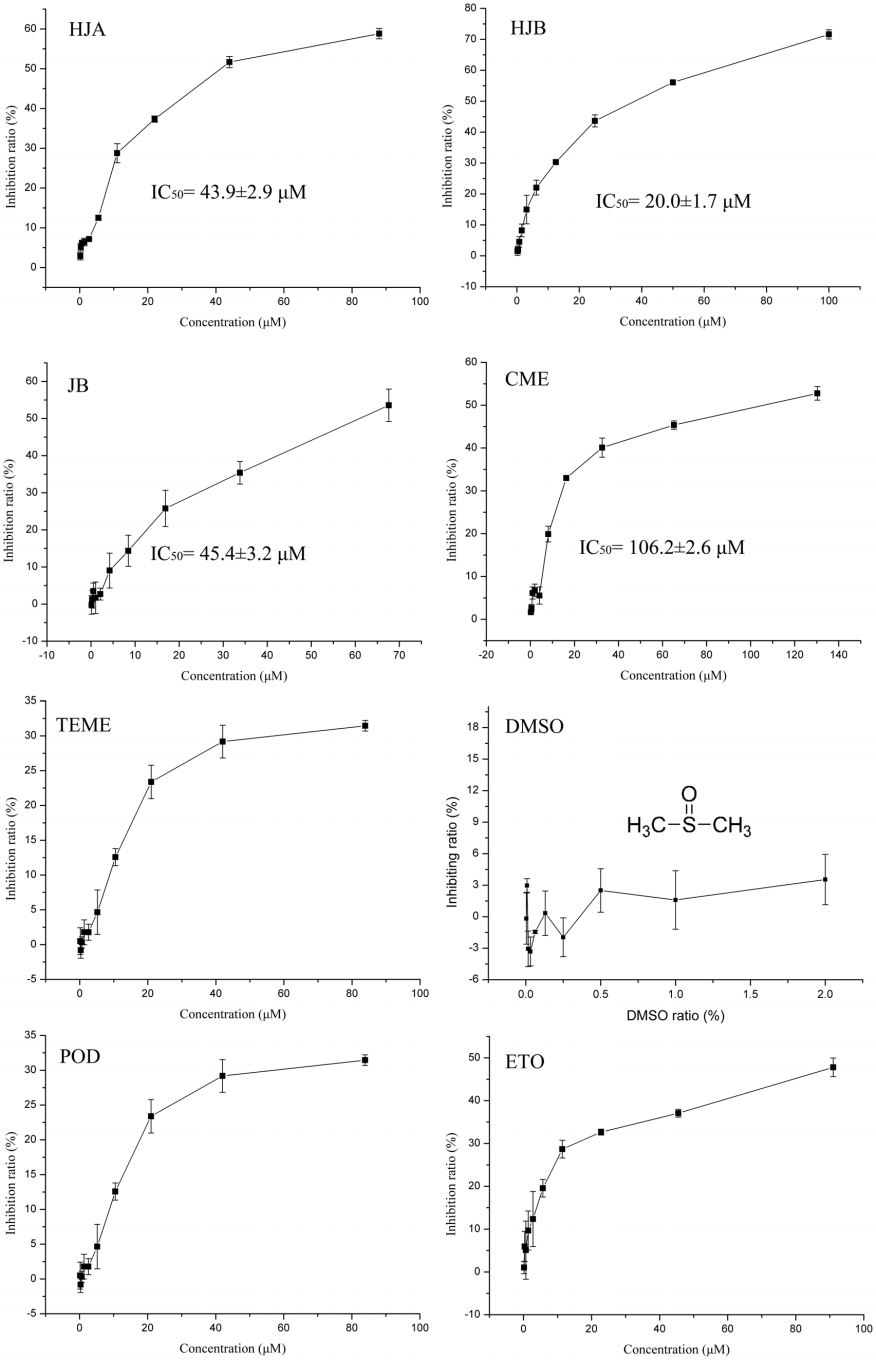
Effect of HJB, HJA, JB, CME, TEME, POD and ETO on K562 cell proliferation. Cells were exposed to the indicated concentrations of arylnaphthalene lignans and incubated for 48± SD of three independent experiments, where each sample was tested in at least triplicate.

To test the cytotoxicity of different doses of HJA, HJB, JB, CME and TEME on human leukemia K562 cells, MTT assay were performed. As shown in Figure 2, arylnaphthalene lignans inhibited the viability of K562 cells to different degree in a dose-dependent manner. After treatment for 48 h, the average 50% inhibition concentrations (IC_50_) of HJB, HJA, JB and CME were 20, 43.9, 45.4 and 106.2 μM, respectively. The IC_50_ of TEME, POD and etoposide (ETO) could not be calculated within the tested concentrations. We also investigated the cytotoxicity of the five arylnaphthalene lignans on HL-60, L1210 and P388D1 cell lines. The results showed that HJB exhibited the most cytotoxicity within these cell lines, with an average IC_50_ ranging from 3.9 to 26.2 μM (see Figure S1–S3). The sequence of the strength of cytotoxicity of the arylnaphthalene lignans was thus deduced to be HJB > HJA > JB > CME > TEME.

To further evaluate the structure-activity relationship, the cytotoxicity of another 10 lignans, derived from *J. procumbens*, was investigated in human colon cancer HCT-8 and human hepatocellular carcinoma Bel-7402 cell lines (Table S1 and Figure S4). Similar to the results obtained with the K562 cell line, HJB and HJA were again found to exhibit the most cytotoxicity, while CME and TEME were only mildly cytotoxic, when each was compared to a vehicle-treated control.

### Arylnaphthalene lignans inhibit SOD activity of leukemia K562 cells

**Figure 3:**
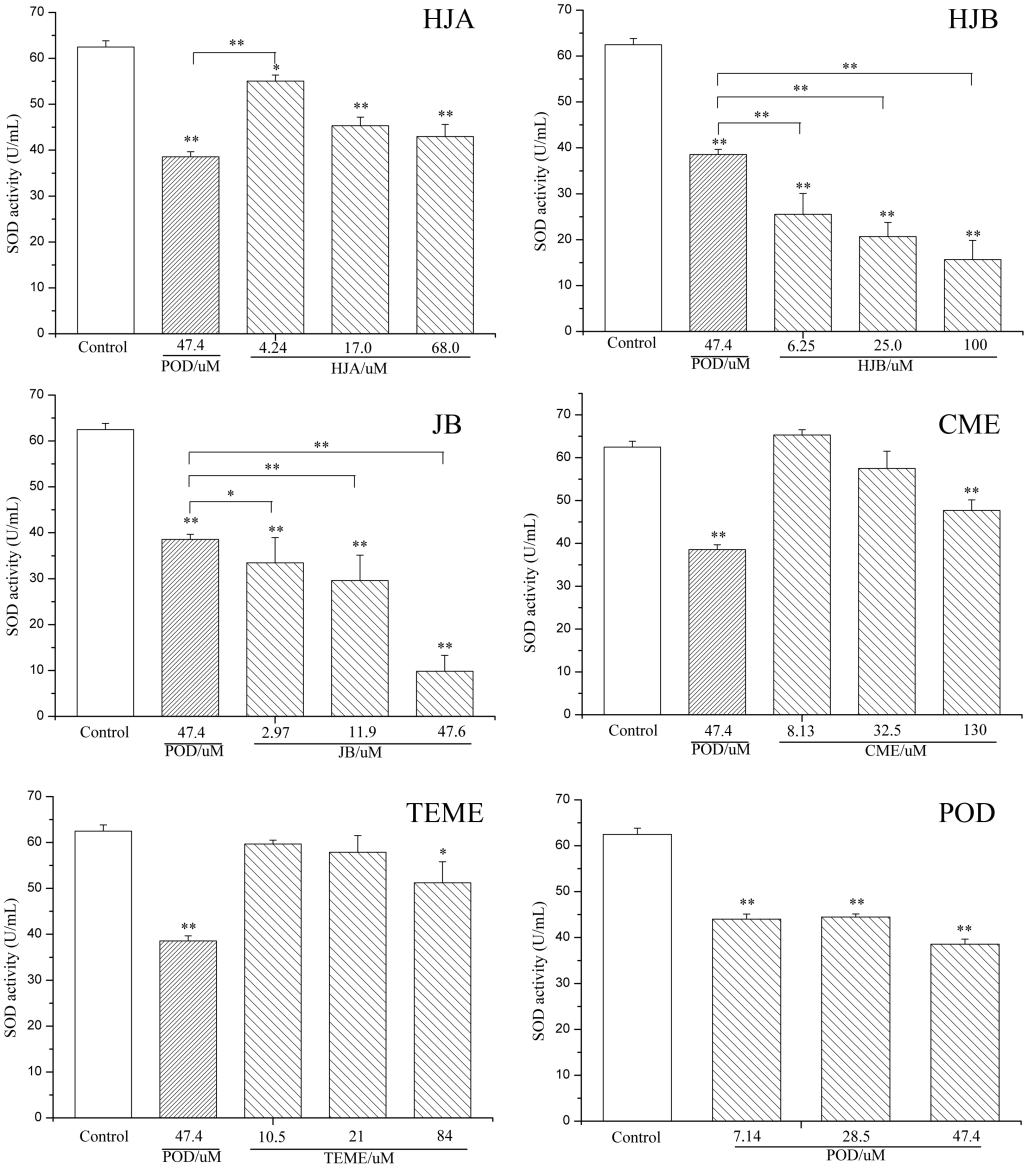
Effect of arylnaphthalene lignans on SOD activity in K562 cells. K562 cells were treated with of the indicated concentrations of HJA, HJB, JB, CME, TEME and POD for 48± SD of five independent experiments; **P<0.01 compared with control group.

To test whether arylnaphthalene lignans altered redox system homeostasis in leukemia K562 cells, superoxide dismutase (SOD) activity, which is involved in the removal of ROS, was measured. Compared with the vehicle-treated control, the activity of SOD in leukemia K562 cells decreased significantly in response to increasing dose of either HJB or JB (Figure 3) while for CME and TEME, only high doses showed inhibitory activity. The sequence of SOD activity inhibition by decreasing strength was HJB > JB > HJA > CME > TEME. Specifically, the SOD activity of cells treated with HJB decreased 59.1, 66.9 and 74.9% at dose of 12.5, 25 and 100 μM, respectively; while with JB it decreased 46.4, 52.6 and 84.3% at 3, 11.9 and 47.6 μM, respectively.

### HJB, HJA and JB increase the proportion of subG0 phase K562 cells

**Figure 4:**
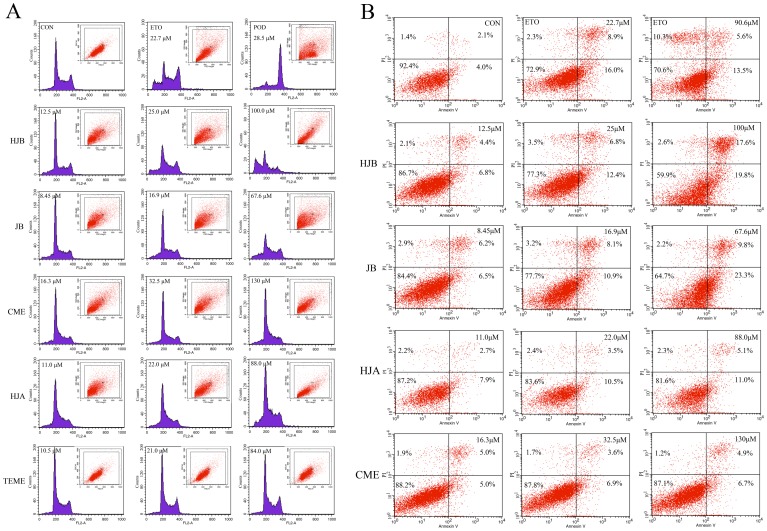
Effect of arylnaphthalene lignans on apoptosis in K562 cells. (A) The apoptosis rate and changes in the cell cycle of K562 cells treated with arylnaphthalene lignans at the indicated doses for 24 h were analyzed by PI staining and subsequent flow cytometry. Representative data are shown. (B) Apoptosis was further analyzed by annexin V/PI staining and flow cytometry of K562 cells treated with the indicated concentrations of HJA, HJB, JB, CME and ETO for 48 h. Representative FACS scatter-grams are shown. All results are representative of three independent experiments.

Next, the effects of the five arylnaphthalene lignan lactones on the cell cycle were analyzed by flow cytometry. It has previously been demonstrated that POD arrests cell development at metaphases. In agreement with this data, K562 cells treated with 28.5 μM POD were arrested at metaphase. Indeed, the percentage of cells in metaphase significantly increased up to 94.1% compared with 6.99% in the vehicle-treated control group (Table 1, Figure 4A). In contrast, treatment of K562 with HJA, HJB, JB and ETO for 48 h dose-dependently increased the proportion of cells in the subG0 phase of the cell cycle while cells in the G2/M phase were barely detected. CME and TEME, however, did not significantly affect the subG0 phase K562 cells (*P*>0.05). Specifically, the percentages of apoptotic K562 cells following exposure to 12.5, 25 and 100 μM HJB were 9.2, 13.6 and 30.5%, respectively, while those of K562 cells exposed to 67.6 μM JB and 100 μM HJB were 14.6 and 30.5%, respectively.

**Table 1:** The apoptosis rate and cell cycle analysis of K562 cells treated with arylnaphthalene lignans for 24

Group	Concentration (μM)	Apoptosis (%)	G0/G1 (%)	S (%)	G2/M (%)
CON	---	2.9±0.2	35.5±2.59	57.5±4.8	7.0±0.3
ETO	22.7	15.3±1.5	19.5±2.0	72.5±5.2	8.0±0.4
POD	28.5	7.9±0.9	5.8±0.8	0.2±0.0	94.1±5.8
HJB	12.5	9.2±1.1	47.6±3.7	44.4±3.7	8.0±0.4
HJB	25.0	13.6±1.5	31.3±3.1	60.6±4.4	8.0±0.4
HJB	100	30.5±2.4	24.1±2.7	75.9±5.3	0.1±0.0
JB	8.4	7.5±0.8	48.5±3.7	47.2±3.9	4.3±0.2
JB	16.9	9.9±1.1	42.3±3.3	53.1±3.7	4.6±0.2
JB	67.6	14.6±1.3	27.4±2.6	64.6±4.1	8.0±0.2
CME	16.3	5.2±1.1	46.2±3.6	52.1±3.7	1.7±0.1
CME	32.5	5.8±0.6	48.5±3.0	45.8±3.3	5.7±0.1
CME	130	7.0±0.9	45.6±3.5	54.4±3.6	0.1±0.0
HJA	11.0	6.0±0.5	44.3±3.6	55.7±3.4	0.1±0.0
HJA	22.0	7.4±0.5	40.9±4.0	59.1±4.5	0.0±0.0
HJA	88.0	8.7±0.5	35.3±2.9	63.6±4.3	1.1±0.0
TEME	10.5	2.8±0.3	40.4±3.5	57.5±3.3	2.1±0.1
TEME	21.0	2.9±0.4	38.7±3.8	54.7±3.5	6.5±0.0
TEME	84.0	3.4±0.4	36.7±2.7	55.3±3.5	8.0±0.1

These data are representative of three independent experiments.

### Effect of arylnaphthalene lignans on apoptosis in K562 cells

The externalization of phosphatidylserine (PS) precedes the loss of membrance integrity which accompanies later stages of cell death induced by either apoptosis or necrosis. Staining with FITC Annexin V, with detects PS, is typically used in conjunction with a vital dye such as propidium iodide (PI) and subsequent analysis by flow cytometry to assess apoptosis. Viable cells with intact membranes exclude PI, whereas the membranes of dead and damaged cells are permeable to PI. As a result, viable cells are negative for both FITC Annexin V and PI; early apoptotic cells are FITC Annexin V positive and PI negative; and late apoptotic or dead cells are both FITC Annexin V and PI positive.

Using this approach, we clearly showed a dose-dependent apoptotic effect of arylnaphthalene lignans in K562 cells (Figure 4B). Indeed, treatment of cells with HJA, HJB, JB and CME at different doses for 48 h resulted in a shift from a population of largely live cells in the vehicle-treated control to populations with increasing proportions of early and late apoptotic cells with little change in the dead cell population (Figure 4B). More specifically, the positive control, ETO, induced apoptosis at a concentration of 22.7 μM (the viable cell population was 72.9%) but it did not increase when its dose was increased to 90.6 μM (the viable cell population was 70.6%). Meanwhile, the apoptotic percentages of K562 cells exposed to HJA, HJB, JB and CME increased in a dose-dependent manner. For example, the early apoptotic percentages of K562 cells exposed to 12.5, 25, and 100 μM HJB were 6.8, 12.4 and 19.8%, respectively; while the viable cell population was 86.7, 77.3 and 59.9%, respectively. The late apoptotic percentages of K562 cells exposed to 8.4, 16.9, and 67.6 μM JB were 6.2, 8.1 and 13.8%, respectively; while the viable cell population was 84.4, 77.7 and 76.2%, respectively.

### Caspase-3 activity assay

The caspase family of cysteine proteases plays a key role in apoptosis and inflammation. Caspase-3 is a key protease that is activated during the early stages of apoptosis and, like other members of the caspase family, is synthesized as an inactive pro-enzyme that is processed in cells undergoing apoptosis by self-proteolysis and/or cleavage by another protease [36]. Active caspase-3, a marker for cells undergoing apoptosis, consists of a heterodimer of 17 and 12 kDa subunits which is derived from the 32 kDa pro-enzyme. Active caspase-3 proteolytically cleaves and activates other caspases, as well as relevant targets in the cytoplasm, e.g., D4-GDI and Bcl-2, and in the nucleus (e.g. PARP). This antibody has been reported to specifically recognize the active form of caspase-3 in human and mouse cells.

**Figure 5:**
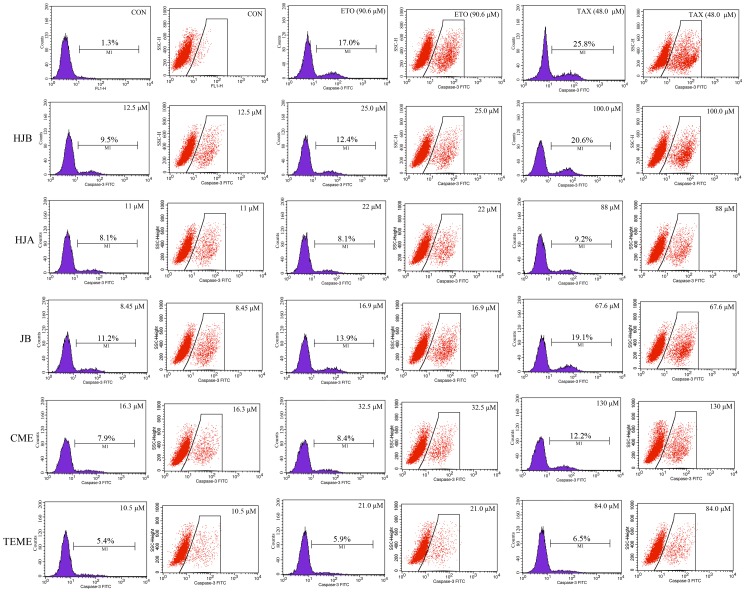
Effects of arylnaphthalene lignans on caspase-3 activity in K562 cells. Active caspase-3 expression was detected by flow cytometry in K562 cells after treatment with the indicated concentrations of HJA, HJB, JB, CME, TEME, ETO and TAX for 48 h. The results are representative of three independent experiments.

As shown in Figure 5, among the tested compounds, HJA, HJB, JB and CME treatment induced caspase-3 activation in K562 cells in a dose-dependent manner. For example, while untreated cells were primarily negative (1.3%) for active caspase-3, treatment with 12.5, 25 and 100 μM HJB increased the percentages of active caspase-3 positive cells to 9.5, 12.4 and 20.6%, respectively. Similarly, the percentages of active caspase-3-positive cells were increased 11.2, 13. 9 and 19.1% when treated with 8.4, 16.9 and 67.6 μM JB, respectively.

## Discussion

Few studies have explored the effects of arylnaphthalene lignans on the human leukemia K562 cell line. Vasilev *et al*. first studied the cytotoxicity and apoptotic effects of JB on K562 cell line [37]. Later, Ionkova *et al*. reported a rare medicinal plant *Linum narbonense* that showed good cytotoxicity in tumor cells and provided a tool for the biotechnological production of JB [38]. However, the detailed effects of other arylnaphthalene lignans such as HJB, CME and TEME, on the leukemia K562 cell line have not been investigated. Moreover, the apoptosis pathways exploited as well as analysis of structure-activity relationships have not yet been illustrated.

In this study, the cytotoxicity of new arylnaphthalene lignans extracted from *J. procumbens*, HJA, HJB, JB, CME and TEME, were tested on the tumor cell lines K562, HL-60, L1210 and P388D1. Of note, these compounds share the same parent nucleus but harbor different substituent groups at the 1, 3′, 4′ and 6′ positions that we hypothesized could underlie variances in activity. We found that the IC_50_ values of HJB in K562, HL-60 and P388D1 cell lines were lower than those of HJA and JB, as determined by MTT assay, indicating that HJB may be a stronger antiproliferative agent than the other tested arylnaphthalene lignans. This result was further validated by the SOD activity assay, cell-cycle analysis and Annexin V/PI staining. In agreement with these findings, we have previously reported that the oral absolute bioavailability of HJB is better than that of HJA (36.0 ± 13.4%) [39] and CME (3.2 ± 0.2%) [40]. Indeed, the sequence of the bioavailability was HJB > JB > HJC > HJA > CME.

ETO, a topoisomerase II inhibitor, is extensively used in the treatment of leukemia. However, K562 cells are known to be less sensitive to ETO than other cell lines [41]. Jiang et al used flow cytometry to measure the apoptosis rate induced by continuous exposure to ETO (10 μM) in K562 cells and found that apoptosis was barely detected 24 h after exposure to ETO [42]. In this study, The IC_50_ of TEME, POD and ETO were not determined within the tested concentrations. Moreover, the cytotoxicity of either POD or ETO on SOD activity was not significant when the concentration was less than 20 μM. In addition, the percentages of cells in both early and late apoptosis when the concentration of ETO was 90.6 μM were lower than those of cells treated with 100 μM HJB. In contrast, HJB, HJA and JB consistently showed greater cytotoxicity, including strong anti-proliferative and pro-apoptotic effects at low doses, suggesting that these compounds might be more effective than ETO in the treatment of leukemia.

In addition to the dose-dependent induction of apoptosis, HJB and JB also impaired SOD activity. SOD is the first line of defense against mitochondria-produced ROS which can protect cells from peroxidation injury induced by transferring one radical (O_2_^−^) to the next (H_2_O_2_) [43]. In this study, the activity of SOD decreased significantly in K562 cells after treatment with different concentrations of HJB and JB, suggesting that these compounds might elicit their cytotoxicity through the impairment of ROS removal.

Caspase-3 is a key effecter molecule of both extrinsic and intrinsic apoptosis. It cleaves a number of cellular proteins, leading to apoptosis [44]. Our study showed that HJB and JB significantly raised the enzymatic activity of caspase-3 in a dose-dependent manner suggesting that these compounds induced apoptosis in K562 cells. Notably, the activity of 100 μM HJB was equal to that of 48 μM paclitaxel (TAX) and 90.6 μM ETO. We thus concluded that arylnaphthalene-induced apoptosis in K562 cells occurred via a caspase-dependent pathway.

Taken together, the sequence of anti-proliferative activity was HJB > HJA > JB > CME > TEME. To explain the differences in anti-proliferative potency of the compounds, structure-activity relationships were examined. First, we found that the substituent at C-1 of the parent nucleus can affect its therapeutic effects. Specifically, a hydroxyl correlated with significantly increased anti-tumor activity while a methoxyl correlated with its decrease. Second, a hydroxyl substituent at C-6′ also correlated with increased anti-tumor activity of the parent nucleus. Third, methylenedioxy between C-3′ and C-4′ correlated with deceased anti-tumor activity compared with methoxyl at the two positions. Of note, it has previously been reported in a structure-activity relationship study that di- and tetrahydronaphthalenes with a trans-lactone and a β-hydroxyl at C-4 have high therapeutic indices in tests for anti-neoplastic and anti-viral effects [45]. Together with our new data, this information could be used to select for arylnaphthalenes with likely high anti-tumor activity based on their structure.

Furthermore, diphyllin, a typical arylnaphthalene lignan isolated from the plant *J. procumbens* (*Acanthaceae*), has been found to have a wide spectrum of biological effects, such as cytotoxicity [19], [20], antimicrobial [24] and antiviral [25] activities. As shown in Figure 1, the structure of HJB is similar to that of diphyllin. The only one difference is the substituted position of hydroxyl, i.e., the hydroxyl substituted at C-1 (diphyllin) translated to position C-6′ (HJB). Therefore diphyllin and HJB, and compounds that bare similar structure, could be good lead compounds for anti-cancer drug design and development.

## Conclusion

This study systematically investigated the anti-tumor activity of five arylnaphthalene lignans isolated from *J. procumbens*. We found that HJB, HJA and JB significantly induced apoptosis in K562 cells via the activation of a caspase-dependent pathway. Structure-activity relationship analysis indicated that hydroxyl substitution at C-1 and C-6′ significantly increase the anti-tumor activity of arylnaphthalene lignans while a methoxyl at C-1 results in a significant decrease. Structure-activity differences could be used to select for arynaphthalenes with superior anti-cancer function for further testing and eventual drug development.

## Materials and Methods

### Materials

Reference standards of POD, ETO and TAX were purchased from Sigma (St. Louis, MO, USA). HJA, HJB, JB, CME, TEME, neojusticidin A, isodiphyllin, Taiwanin C, neesiinoside C, 6′-hydroxy azizin, 4′-demethylchinensinaphthol methyl ether, Diphyllin-1-O-*β*-D-apiofuranoside, ciliatoside C, Justicidinoside C and Justicidinoside B were isolated from *J. procumbens* and identified by UV, IR, ESI-MS, ^1^H and ^13^C-NMR [28], [29]. The purity of the chemicals was greater than 95% as determined by normalization of the peak areas detected by HPLC-UV. The Cycle TEST PLUS DNA Reagent kit, FITC Active Caspase-3 Apoptosis Kit and FITC Annexin V Apoptosis Detection Kit were purchased from BD Pharmingen (San Diego, CA, USA). The SOD Activity Assay Kit was purchased from Nanjing Jiancheng Bioengineering Institute (Nanjing, China). Other reagents were purchased from Sigma (St. Louis, MO, USA).

### Cell cultures

Human leukemia K562, human promyelocytic leukemia HL-60, mouse lymphocytic leukemia L1210, P388D1 mouse macrophage, human colon cancer HCT-8 and human hepatocellular carcinoma Bel-7402 cell lines were obtained from the Cancer Institute & Hospital, Chinese Academy of Medical Sciences, and the original commercial source was the American Type Culture Collection (ATCC, Manassas, VA, USA). The cell lines were cultured in RPMI-1640 (GIBCO BRL, Grand Island, NY, USA) containing 10% heat-inactivated fetal bovine serum, 100 U/ml penicillin, and 100 U/ml streptomycin. Cells were maintained at 37°C in an atmosphere of 5% carbon dioxide/95% air.

### Cell viability assay

Cytotoxicity was determined using a modified MTT colorimetric assay [2]. HJA, HJB, JB, CME and TEME were dissolved in dimethylsulfoxide (DMSO; final concentration 0.1% (v/v)). DMSO (0.1%) was used as the control. Briefly, cells were seeded in 96-well plates (Nunc, Roskilde, Denmark) at 1×10^5^ cells/ml and treated for 48 h with different concentrations of the test agent, as indicated. Next, 20 μl MTT (5 mg/ml) was added to each well and cells were incubated at 37°C for a further 4 h. Then, 100 μl 10% SDS-5% isopropanol-0.012 M HCl was added and cells were incubated overnight at 37°C. The absorbance of each well was measured at 570 nm in a Multiscan photometer (BioTek, μQuant, USA). The IC_50_ was calculated by the following equation: IC_50_ = Log^−1^(Xm-I(∑p-(3-Pm-Pn)/4)), whereas Xm represents the logarithm of the maximum concentration, I represents the dilution factor, Pm represents the maximum inhibition rate, and Pn represents the minimum inhibition rate.

### Measurement of SOD activity

The activity of SOD was detected by its ability to reduce cytochrome *c*, which causes an increase in absorbance at 550 nm. To do this, K562 cells were plated in 96-well plates at 1×10^5^ cells/ml and allowed to attach for 24 h. Cells were then treated with different doses of the indicated arylnaphthalene or vehicle control. After 48 h, K562 cells were thawed three times at a temperature of −80°C. The supernatant was collected and used to measure intracellular SOD activity at 550 nm by using an automatic microplate reader.

### Cell-cycle analysis

K562 cells were seeded at a density of 1×10^5^ cells/ml in 12-well plates and incubated with different concentrations of the indicated arylnaphthalene for 48 h. After exposure, 10^6^ cells were harvested, washed twice with ice-cold PBS, then centrifuged to recover the cell pellet which was subsequently resuspended and fixed with 70% ethanol overnight at 4°C. The suspension was then washed and resuspended in 100 μl PBS containing 100 μg/ml RNase at RT for 10 min followed by staining with 200 μl of 100 μg/ml PI for 20 min in darkness. PI-stained cells were assayed using FACS Canto Becton Dickinson Flow Cytometry and cell cycle distributions were analyzed with the ModFit program (BD, San Diego, CA, USA). All samples were assayed in triplicate, and the fraction of each cell cycle phase was calculated.

### Annexin V/PI staining and flow cytometry analysis

K562 cells were plated in 12-well plates at a density of 5×10^5^ cells/well. The cells were treated with varying concentrations of the indicated arylnaphthalenes or the vehicle (0.1% DMSO) in complete medium for 48 h. At the end of each treatment, cells were collected and the quantitative apoptotic death assay was performed by Annexin V and PI staining (Molecular Probes) following the manufacturer's protocol. Staining was then analyzed immediately by flow cytometry using the FACS (BD, San Diego, CA, USA).

### Caspase-3 activity assay

Caspase 3 enzymatic activity was measured using the caspase 3 activity assay kits (BioVision, USA) according to the manufacturer's instructions. In brief, cells were seeded at a density of 5×10^5^ cells/ml in 12-well slides. After treatment with varying concentrations of the indicated Arylnaphthalene or the vehicle (0.1% DMSO) for 12 h, cells were collected and washed twice with ice-cold PBS, then resuspended in Cytofix/Cytoperm solution at a concentration of 1×10^5^ cells/50 μl. The cells were incubated for 20 min on ice and then washed twice with Perm/Wash buffer at a volume of 0.5 ml buffer/1×10^6^ cells at RT. The cells were then incubated with 20 μl FITC anti-active caspase-3/1×10^6^ cells for 30 min at RT. After that, each sample was washed in 1 ml Perm/Wash buffer, and then resuspended in 0.5 ml Perm/Wash buffer and analyzed by flow cytometry using the FACS (BD, San Diego, CA, USA).

### Statistical analysis

All experimental data are shown as means ± S.D. and accompanied by the number of experiments. Analysis was performed using one-way ANOVA followed by Dunnetts post-hoc test, and the values for significant difference were set at *p<0.05 and **p<0.01.

## Supporting Information

Figure S1 **Effect of HJB, HJA, JB, CME, TEME, POD, ETO and TAX on the proliferation of HL-60 cells.** HL-60 cells were exposed to the indicated concentrations of arylnaphthalene lignans and incubated for 48 h, MTT assays were then performed. Data represent the mean ± SD of three independent experiments, where each sample was tested in at least triplicate.
(TIF)

Figure S2 **Effect of HJB, HJA, JB, CME, TEME, POD, ETO and TAX on the proliferation of L1210 cells.** L1210 cells were exposed to the indicated concentrations of arylnaphthalene lignans and incubated for 48 h, MTT assays were then performed. Data represent the mean ± SD of three independent experiments, where each sample was tested in at least triplicate.
(TIF)

Figure S3 **Effect of HJB, HJA, JB, CME, TEME and TAX on the proliferation of P388D1 cells.** P388D1 cells were exposed to the indicated concentrations of arylnaphthalene lignans and incubated for 48 h, MTT assays were then performed. Data represent the mean ± SD from three independent experiments, where each sample was tested in at least triplicate.
(TIF)

Figure S4 **Chemical structures of 10 lignans isolated from** ***J. procumbens*** **, including neojusticidin A, isodiphyllin, Taiwanin C, neesiinoside C, 6′-hydroxy azizin, 4′-demethylchinensinaphthol methyl ether, Diphyllin-1-O-** ***β*** **-D-apiofuranoside, ciliatoside C, Justicidinoside C and Justicidinoside B.**
(TIF)

Table S1 **Cytotoxicity of 14 lignans derived from** ***J. procumbens*** **on the HCT-8 and Bel-7402 cell lines.**
(DOC)
