# Supplementary figures and images for: Evaluation and Structure-Activity Relationship Analysis of a New Series of Arylnaphthalene lignans as Potential Anti-Tumor Agents
Source: PLoS One. 2014 Mar 27;9(3):e93516. doi: 10.1371/journal.pone.0093516 (PMC3968169; doi:10.1371/journal.pone.0093516)

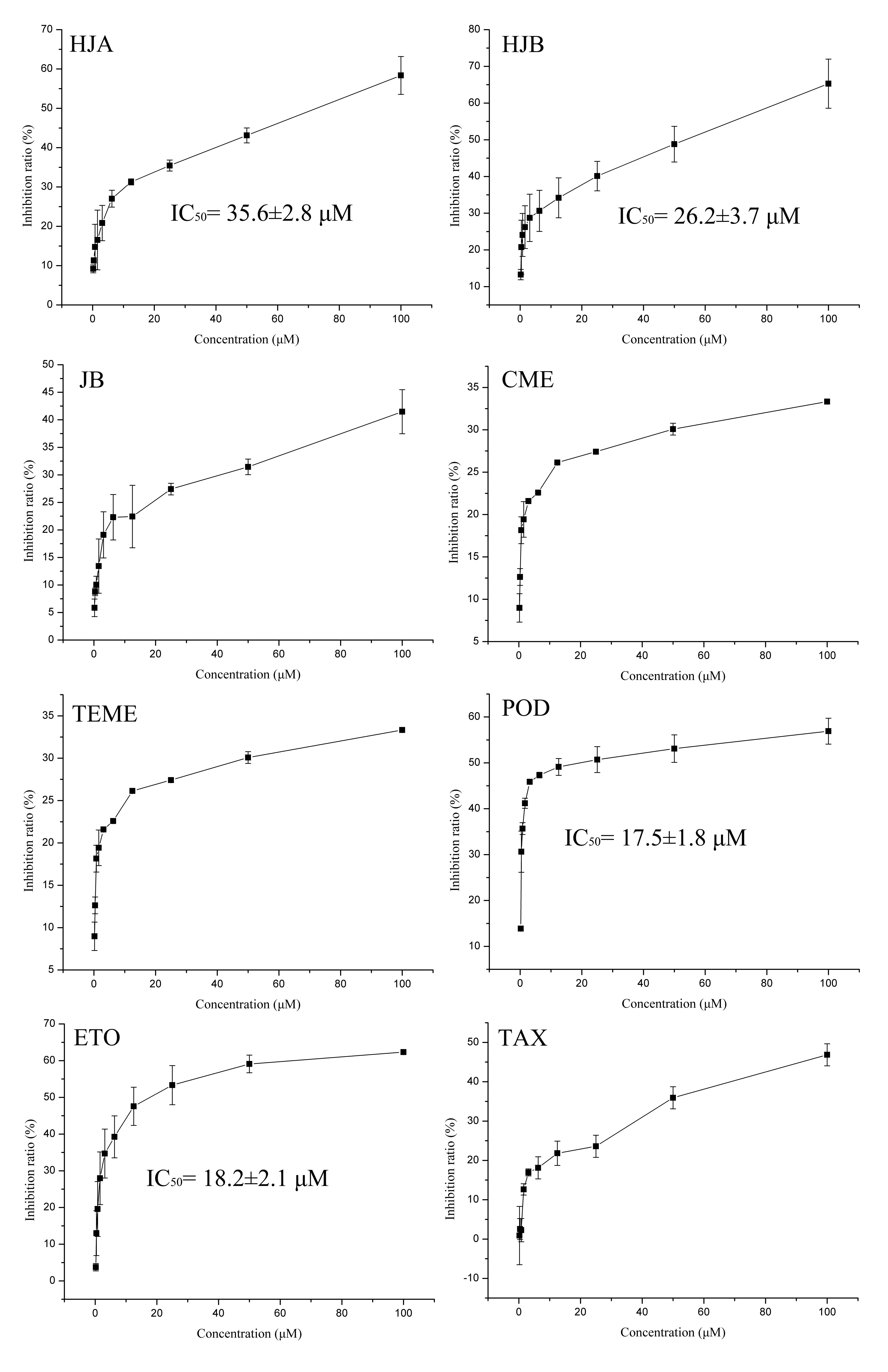

Supplement: Figure S1 — Effect of HJB, HJA, JB, CME, TEME, POD, ETO and TAX on the proliferation of HL-60 cells. HL-60 cells were exposed to the indicated concentrations of arylnaphthalene lignans and incubated for 48 h, MTT assays were then performed. Data represent the mean ± SD of three independent experiments, where each sample was tested in at least triplicate. (TIF) [file pone.0093516.s001.tif]

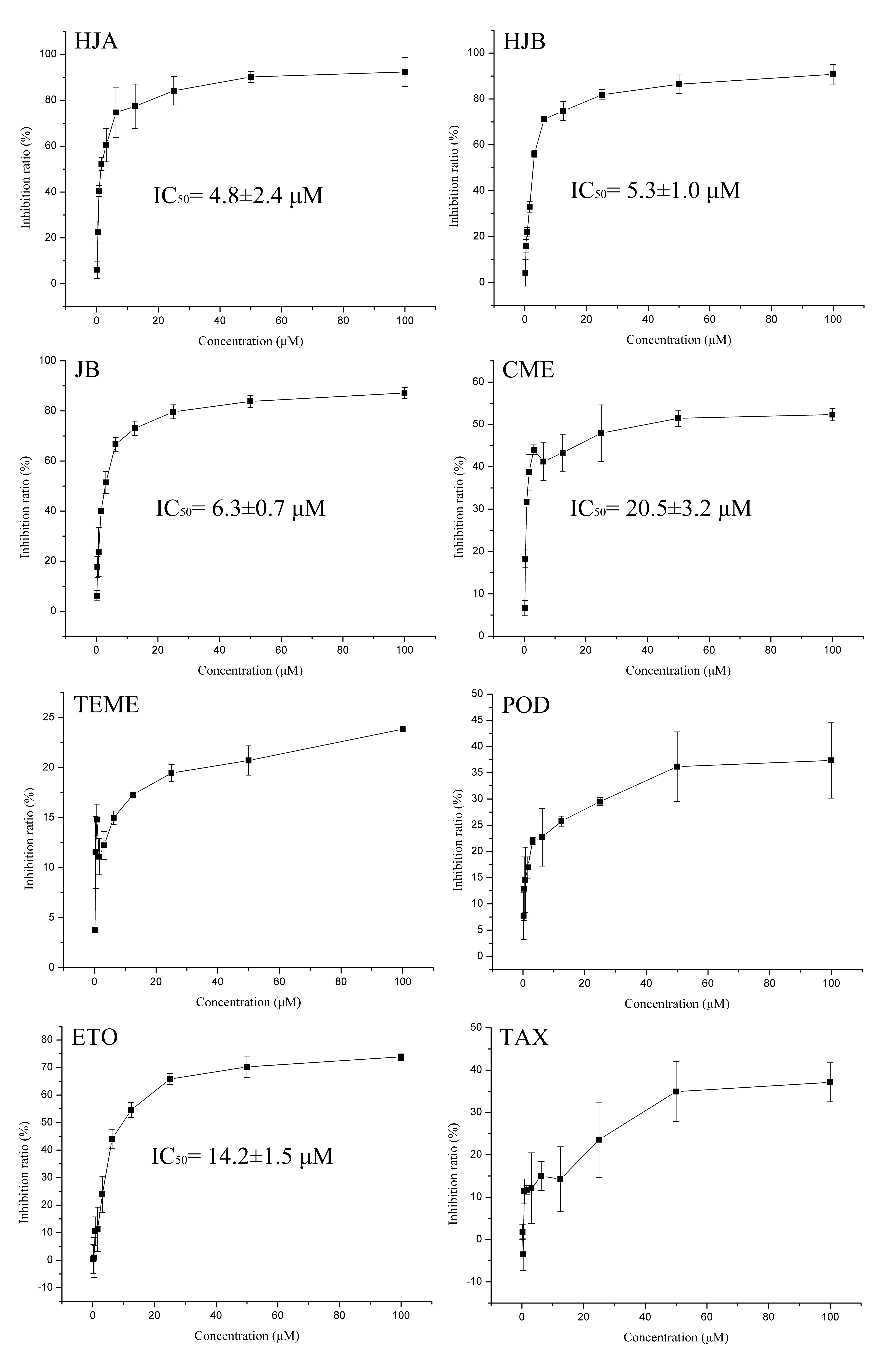

Supplement: Figure S2 — Effect of HJB, HJA, JB, CME, TEME, POD, ETO and TAX on the proliferation of L1210 cells. L1210 cells were exposed to the indicated concentrations of arylnaphthalene lignans and incubated for 48 h, MTT assays were then performed. Data represent the mean ± SD of three independent experiments, where each sample was tested in at least triplicate. (TIF) [file pone.0093516.s002.tif]

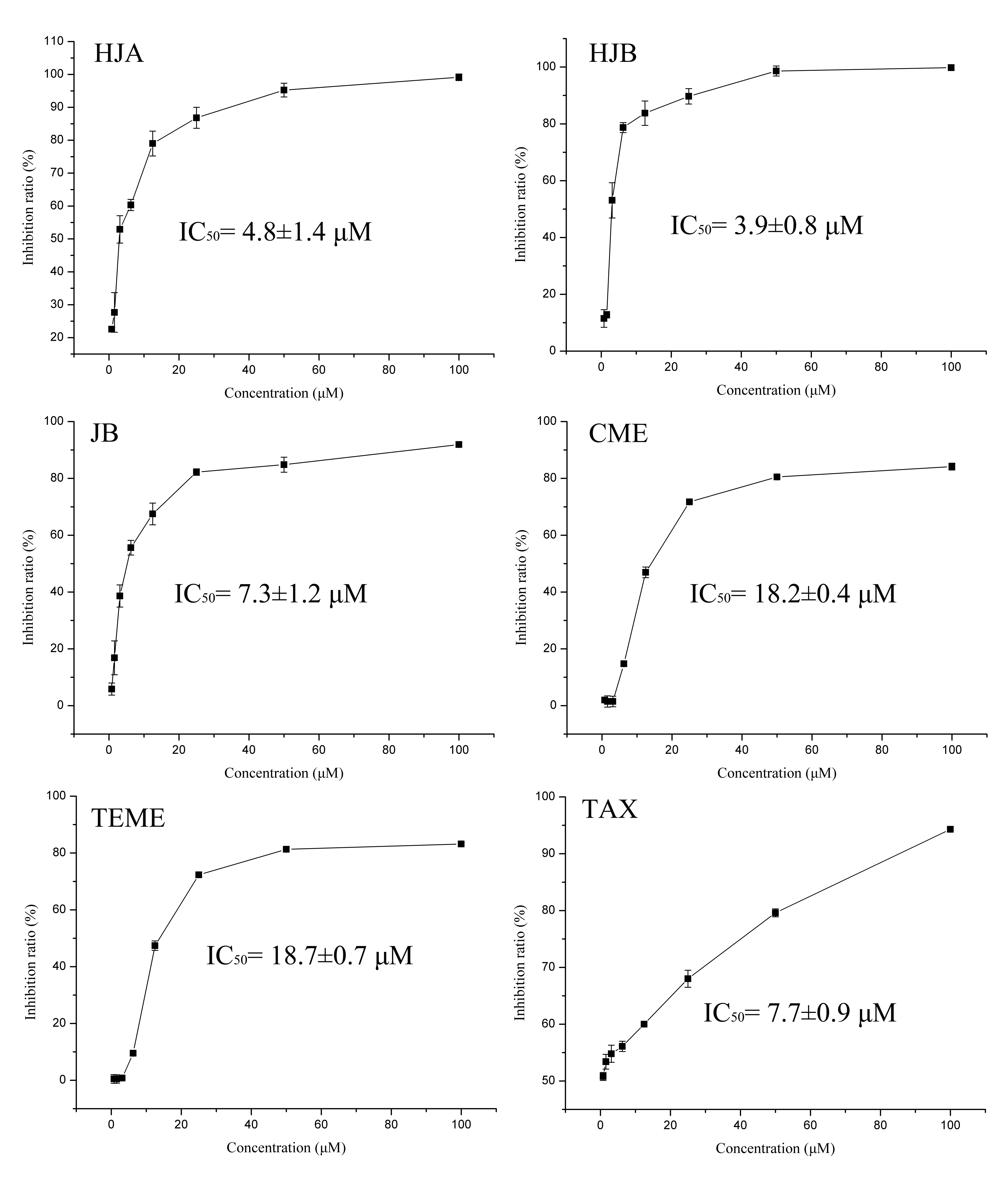

Supplement: Figure S3 — Effect of HJB, HJA, JB, CME, TEME and TAX on the proliferation of P388D1 cells. P388D1 cells were exposed to the indicated concentrations of arylnaphthalene lignans and incubated for 48 h, MTT assays were then performed. Data represent the mean ± SD from three independent experiments, where each sample was tested in at least triplicate. (TIF) [file pone.0093516.s003.tif]

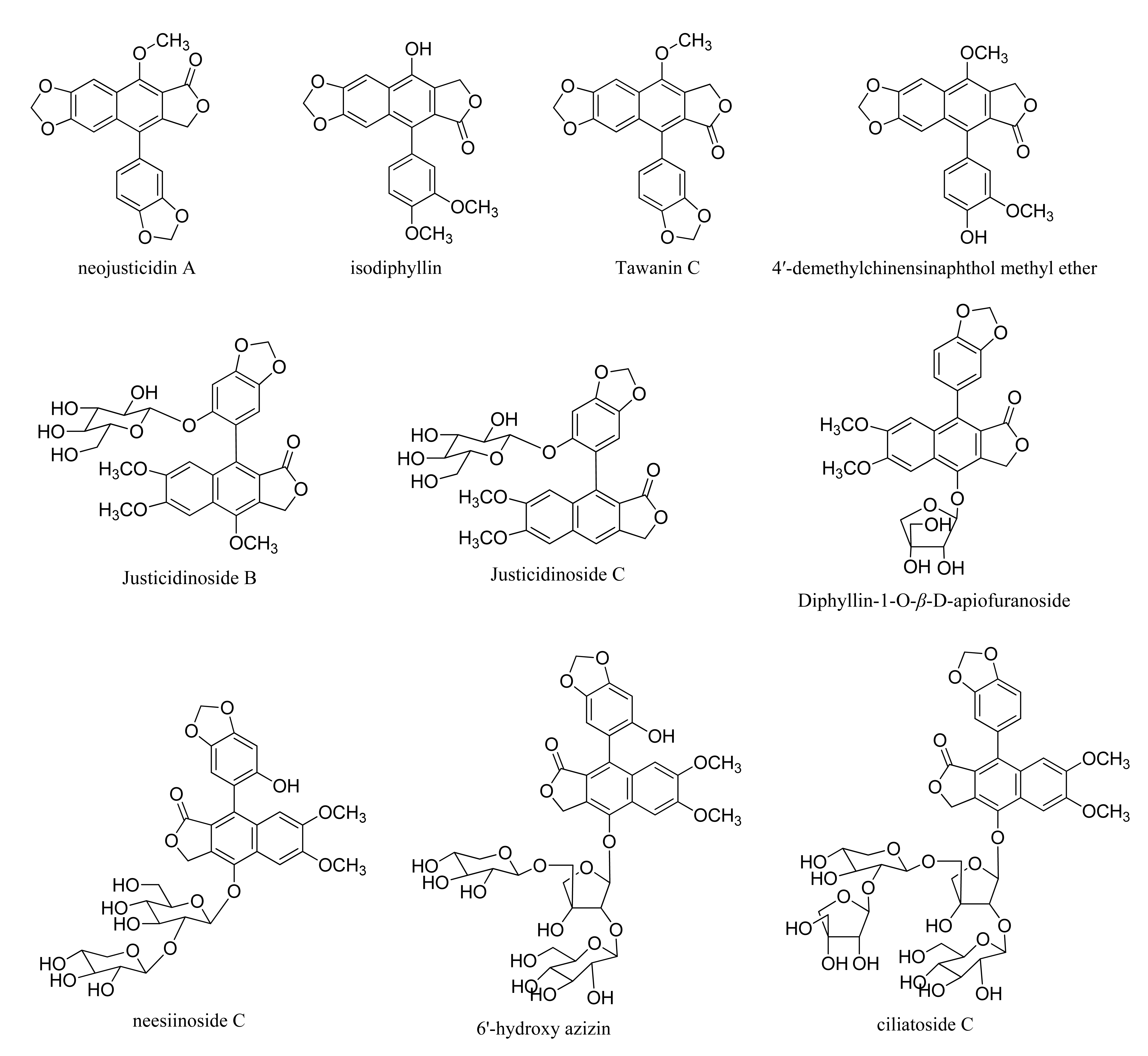

Supplement: Figure S4 — Chemical structures of 10 lignans isolated from J. procumbens , including neojusticidin A, isodiphyllin, Taiwanin C, neesiinoside C, 6′-hydroxy azizin, 4′-demethylchinensinaphthol methyl ether, Diphyllin-1-O- β -D-apiofuranoside, ciliatoside C, Justicidinoside C and Justicidinoside B. (TIF) [file pone.0093516.s004.tif]
