# Supplementary material for: Evaluation and Structure-Activity Relationship Analysis of a New Series of Arylnaphthalene lignans as Potential Anti-Tumor Agents
Source: PLoS One. 2014 Mar 27;9(3):e93516. doi: 10.1371/journal.pone.0093516 (PMC3968169; doi:10.1371/journal.pone.0093516)
Supplement: Table S1 — Cytotoxicity of 14 lignans derived from J. procumbens on the HCT-8 and Bel-7402 cell lines. (DOC) [file pone.0093516.s005.doc]

| Compounds | IC_50_ (μM) | |
| --- | --- | --- |
|  | HCT-8 | Bel-7402 |
| neojusticidin A | 6.6 | 12.0 |
| chinensinaphthol methyl ether (CME) | >20 | >20 |
| 6’-hydroxy justicidin B (HJB) | 1.6 | 2.5 |
| Taiwanin E methyl ether (TEME) | >20 | >20 |
| isodiphyllin | 13.8 | 12.6 |
| 6’-hydroxy justicidin A (HJA) | 1.4 | 2.9 |
| Tawanin C | >20 | 12.5 |
| neesiinoside C | >20 | >20 |
| 6'-hydroxy azizin | >20 | >20 |
| ciliatoside C | >20 | >20 |
| 4′-demethylchinensinaphthol methyl ether | >20 | >20 |
| Diphyllin-1-O-*β*-D-apiofuranoside | 3.2 | 9.8 |
| Justicidinoside C | >20 | >20 |
| Justicidinoside B | >20 | >20 |
